# Adding Chinese Herbal Medicine to Routine Care is Associated With a Lower Risk of Rheumatoid Arthritis Among Patients With Asthma: A Population-Based Retrospective Cohort Study

**DOI:** 10.3389/fphar.2022.895717

**Published:** 2022-08-17

**Authors:** Wei-Chiao Chang, Hanoch Livneh, Wei-Jen Chen, Chang-Cheng Hsieh, Yu-Han Wang, Ming-Chi Lu, How-Ran Guo, Tzung-Yi Tsai

**Affiliations:** ^1^ Department of Chinese Medicine, Dalin Tzuchi Hospital, The Buddhist Tzuchi Medical Foundation, Chiayi, Taiwan; ^2^ Rehabilitation Counseling Program, Portland State University, Portland, OR, United States; ^3^ Graduate Institute of Sports Science, National Taiwan Sport University, Taoyuan, Taiwan; ^4^ School of Post-Baccalaureate Chinese Medicine, Tzu Chi University, Hualien, Taiwan; ^5^ Center of Sports Medicine, Dalin Tzuchi Hospital, The Buddhist Tzuchi Medical Foundation, Chiayi, Taiwan; ^6^ Department of Family Medicine, Dalin Tzuchi Hospital, The Buddhist Tzuchi Medical Foundation, Chiayi, Taiwan; ^7^ Division of Allergy, Immunology and Rheumatology, Dalin Tzuchi Hospital, The Buddhist Tzuchi Medical Foundation, Chiayi, Taiwan; ^8^ School of Medicine, Tzu Chi University, Hualien, Taiwan; ^9^ Department of Environmental and Occupational Health, College of Medicine, National Cheng Kung University, Tainan, Taiwan; ^10^ Department of Occupational and Environmental Medicine, National Cheng Kung University Hospital, Tainan, Taiwan; ^11^ Occupational Safety, Health and Medicine Research Center, National Cheng Kung University, Tainan, Taiwan; ^12^ Department of Nursing, Tzu Chi University of Science and Technology, Hualien, Taiwan; ^13^ Department of Medical Research, Dalin Tzuchi Hospital, The Buddhist Tzuchi Medical Foundation, Chiayi, Taiwan

**Keywords:** asthma, Chinese herbal medicines, rheumatoid arthritis, cohort study, nationwide study, propensity score matching

## Abstract

**Objective:** Due to the shared pathogenesis of asthma and rheumatoid arthritis (RA), patients with asthma were found to have a higher risk of RA. While the benefits and safety of Chinese herbal medicine (CHM) for asthma have been reported, the scientific evidence regarding its effect on RA is limited. This longitudinal cohort study aimed to determine the relation between CHM use and RA risk in patients with asthma.

**Methods:** Using the nationwide claims data, we enrolled 33,963 patients 20–80 years of age who were newly diagnosed with asthma and simultaneously free of RA between 2000 and 2007. From this sample, we utilized propensity score matching to create sets of participants as treatment and control groups, which comprised 13,440 CHM users and 13,440 non-CHM users. The incidence rate and hazard ratio (HR) for RA between the two groups were estimated at the end of 2013. A Cox proportional hazards model was constructed to examine the impact of the CHM use on the risk of RA.

**Results:** The cumulative incidence of RA was substantially lower in the CHM user group. In the follow-up period, 214 patients in the CHM user group (1.92 per 1,000 person-years) and 359 patients in the non-CHM user group (2.92 per 1,000 person-years) developed RA (adjusted HR = 0.63, 95% confidence interval: 0.54–0.75). Of the commonly-prescribed formulae, nine CHM products were associated with a lower RA risk: Xiao-Qing-Long-Tang, Ma-Xing-Gan-Shi-Tang, Ding-Chuan-Tang, Xin-Yi-Qing-Fei-Tang, Bei Mu, Jie Geng, Xing Ren, Da Huang, and San Chi.

**Conclusion:** This study found that patients with asthma who received CHM treatment, in addition to the conventional therapy, had a lower risk of RA. Use of CHM treatment may be integrated into conventional therapy to reduce subsequent RA risk among asthma patients.

## Introduction

Chronic inflammation processes are involved in a wide variety of mental and physical health conditions that result in high rates of morbidity and mortality (Furman et al., 2019). For example, asthma is a chronic inflammatory disorder of the airway that includes both airflow limitation and hyper-responsiveness, manifesting with clinical symptoms of wheezing, dyspnea and chest tightness (Yeh and Schwartzstein, 2009). Asthma affects an estimated 300 million individuals worldwide, a number estimated to exceed 400 million by 2025, thus posing an enormous burden to both the patient and the healthcare system (Partridge, 2007; Gruffydd-Jones et al., 2019). In the United States, for example, the annual medical cost of asthma during 2008–2013 was estimated at about $50.3 billion; the total annual societal costs would rise to nearly $82 billion after adding the intangible costs of deterioration in quality-of-life and premature mortality (Nurmagambetov et al., 2018).

Asthma has been recognized as a trigger for several debilitating diseases, in addition to its enormous economic burden. Recent evidence indicates that asthma may put patients at a higher risk of autoimmune rheumatic diseases, including rheumatoid arthritis (RA). A meta-analysis of 20 studies indicated patients with asthma had almost twice the risk of developing RA as did the general population (Charoenngam et al., 2020). It was suggested that the imbalance of T helper (Th) 1/Th2 cell immunity may play an indispensable role in the pathogenesis of allergic asthma (Rolfes et al., 2017; Charoenngam et al., 2020), and one recent review article indicated that Th17 cells, another T cell lineage distinct from Th1 and Th2 cells and featured by the secretions of IL-17, IL-17F, IL-22, and other cytokines, can provoke autoimmunity *via* promoting tissue inflammation and mobilizing innate immunity, which could provide a connection between two diseases (Rolfes et al., 2017). Worse yet, patients with asthma and concurrent RA may experience a nearly 50% higher in-hospital mortality risk than those with asthma alone (Luo et al., 2018). Such alarming clinical manifestations call for the urgent need to seek out treatments or interventions that can reduce the RA risk while managing asthma.

Chinese herbal medicine (CHM) has shown promising results in treating chronic disorders, including allergic diseases (Chan and Ng, 2020). One recent study found that compared to asthma patients treated with the conventional Western medicine, those treated with a combination of CHM and Western medicine substantially decreased the number of asthma admission by nearly 40% (Liao et al., 2022). Compounds isolated from CHM have been proven to inhibit the inflammation reaction induced by chemical agents and down-regulate the serum level of immunoglobulin E (Yan et al., 2021; Zhu et al., 2021). Meanwhile, some antioxidant and anti-inflammatory medicinal plants were found to be involved in the treatment of rheumatologic conditions (Pan et al., 2011; Yu et al., 2014). Accordingly, in view of the mounting evidence reporting that both asthma and RA may be sparked by the abnormal inflammatory responses (Rolfes et al., 2017; Charoenngam et al., 2020), the use of CHM might be considered while instituting a novel care regimen to prevent or delay the development of RA for asthma patients. To the best of our knowledge, no study has so far investigated the long-term effect of CHM on an initial RA event in patients with asthma. To address this issue, we utilized administrative data to determine whether adding CHM to conventional treatment could lower the incidence of RA among patients with asthma.

## Methods

### Data Source and Selection of Study Population

Research data for this retrospective cohort study were subtracted from the Longitudinal Health Insurance Database (LHID) administered by the Ministry of Health and Welfare of Taiwan. The National Health Insurance (NHI) program was implemented in March 1995 and presently covers more than 99% of Taiwan’s 23 million residents. The LHID 2000 contains data on one million beneficiaries randomly sampled from the Registry for Beneficiaries of the NHI Research Database. The use of multistage stratified systematic sampling ensures no remarkable deviations in the distribution of sex and age between the LHID enrollees and the general population (National Health Insurance Administration, 2016). The claim files include inpatient and outpatient demographics, primary and secondary diagnoses, procedures, prescriptions, and medical expenditures. The Ministry of Health and Welfare manages the claim data and assigns scrambled random identification numbers to beneficiaries to protect their privacy. Accordingly, the Institutional Review Board and Ethics Committee of Buddhist Dalin Tzu Chi Hospital approved this study and waived informed consents for the entire study cohort (Approval Number: B10803015-1).

**FIGURE 1:**
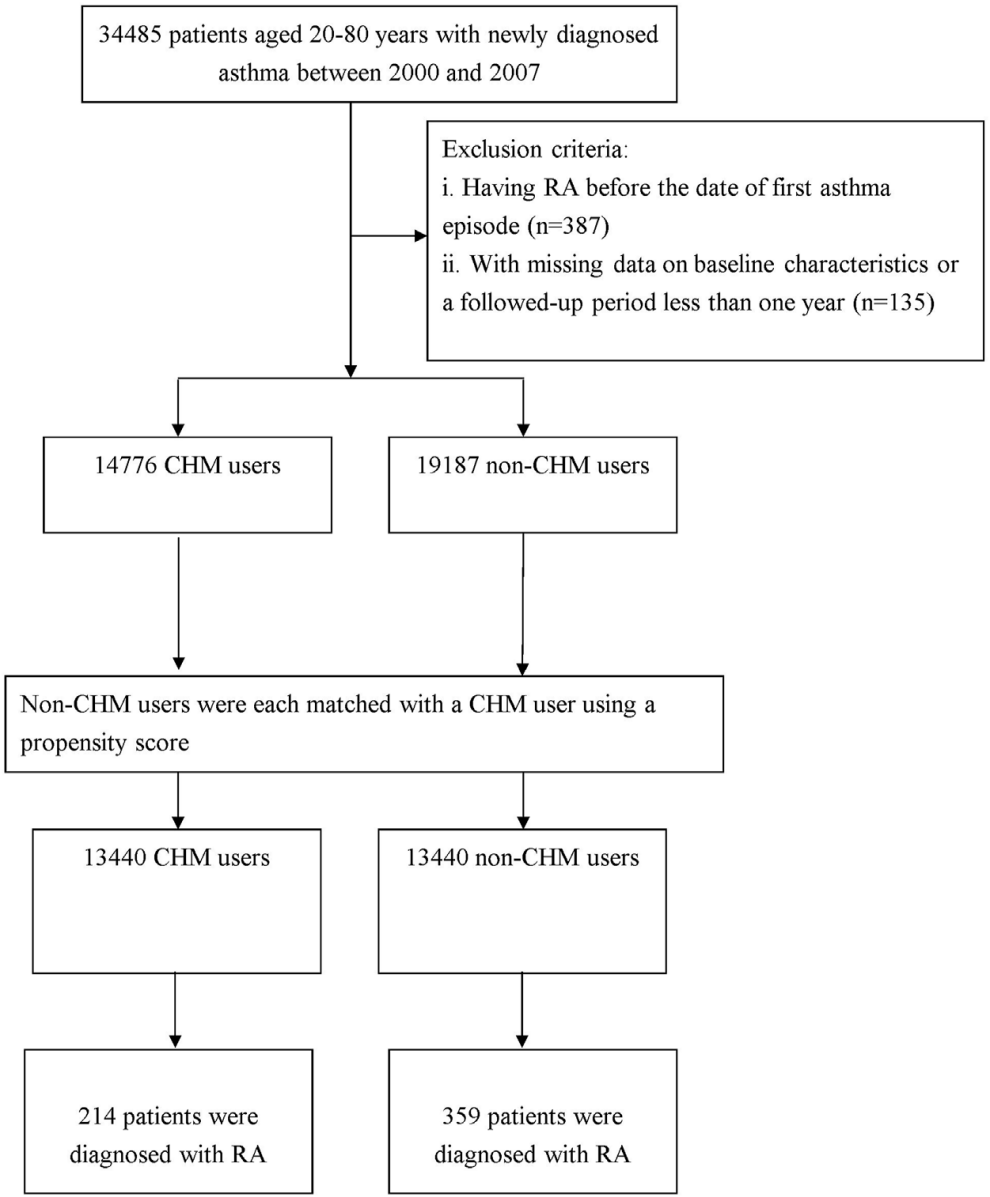
Flowchart showing the method of selecting and following study subjects.

The method used to select the participants is illustrated in Figure 1. Diagnoses in the insurance claim data were coded using the International Classification of Diseases, Ninth Revision, Clinical Modification (ICD-9-CM). From the database, we collected claims data submitted for subjects aged 20–80 years, and they had at least one admission code or three or more outpatient codes for asthma within 365 calendar days between 2000 and 2007 (ICD-9-CM code 493). This algorithm of at least one inpatient visit or three outpatient visits in a 12-months span was often used to define the diseases while using the administrative claims data in from LHID (Wang Y. et al., 2020; Li H.-H. et al., 2021). Overall, we enrolled 34,485 cases with new-onset asthma.

### Outcome of Interest

The study outcome was the first diagnosis with RA incidence. In this work, we applied the catastrophic illness registry to verify the RA event. Only those patients with a catastrophic illness certification for the ICD-9-CM code of 714.0 were deemed RA cases. In Taiwan, insured residents with major diseases (e.g., cancer, autoimmune disease, chronic mental disease, and end-stage renal failure) can apply for a catastrophic illness certificate to be exempt from co-payment. Meanwhile, to establish a temporal link between asthma and RA, we deleted those who had a diagnosis of RA prior to the first asthma admission/visit (*n* = 387) and who were followed for less than 1 year after the cohort entry, or had missing data on baseline characteristics (*n* = 135). Finally, a total of 33,963 qualified patients with asthma were identified.

### Exposure of Chinese Herbal Medicine

Under the NHI program, only certified Chinese medicine physicians are allowed to provide CHM treatment. In accordance with formerly-established method (Li KH. et al., 2021), CHM users were identified as those who received CHM to treat asthma for more than 30 days, whereas those treated for 30 days or less were considered to be non-CHM users. Based on this designation, a total of 14,776 patients were classified as CHM users in this investigation. A non-CHM user group was randomly selected from the remaining enrollees for comparison. For each patient receiving CHM, one non-CHM user was selected *via* one-to-one propensity score matching. The propensity score value, the predicted probability of CHM exposure, was calculated using logistic regression on the basis of patients’ demographics as shown in Table 1, containing age, sex, monthly income, residential area and comorbidities. Every CHM user was randomly matched with a non-CHM user who had the nearest propensity score, where the difference in the score ranged between −0.1 and +0.1 (Austin, 2011). Afterwards, we calculated the person-year (PY) starting from the initiation of CHM usage to correct for immortal time for patients who received CHM (Shariff et al., 2008). The index date of the follow-up period for a non-CHM user was assigned as the date of the first asthma diagnosis, whereas the index date of the follow-up period for a CHM user was assigned as the first date of the commencement of CHM prescription. All patients were followed up to the end of 2013 for the occurrence of RA incidence. The follow-up time, in PY, was determined by calculating the time interval from the index date to the earliest of the following end points: diagnosis of RA, withdrawal from the insurance (mostly due to death), and 31 December 2013.

**TABLE 1:** Patient demographic data and comorbidities.

	Total patients	Non-CHM users *n* = 13,440 (%)	CHM users *n* = 13,440 (%)	Standardized difference
Age (years)				0.0001
Mean (SD)	52.10 (16.31)	52.02 (16.89)	52.18 (15.71)	
Age				0.005
≤50 years	11,931 (44.4)	5,979 (44.5)	5,952 (44.3)	
>50 years	14,949 (55.6)	7,461 (55.5)	7,488 (55.7)	
Sex				0.004
Male	11,652 (43.3)	5,666 (42.2)	5,686 (42.3)	
Female	15,228 (57.7)	7,774 (57.8)	7,754 (57.7)	
Monthly income (in NTD)				0.0009
≤17,800	12,596 (46.9)	6,284 (46.8)	6,312 (47.0)	
17,881–43,900	13,279 (49.4)	6,664 (49.6)	6,615 (49.2)	
≥43,901	1,005 (3.7)	492 (3.7)	513 (3.8)	
Residential area				0.0004
Urban	15,112 (56.2)	7,588 (56.5)	7,524 (56.0)	
Suburban	4,251 (15.8)	2059 (15.3)	2,192 (16.3)	
Rural	7,517 (28.0)	3,793 (28.2)	3,724 (27.7)	
CCI				0.01
Mean (SD)	6.32 (9.09)	6.24 (9.05)	6.41 (9.01)	

CCI: Charlson-Deyo Comorbidity Index; CHM: Chinese herbal medicine; NTD: New Taiwan Dollar; SD: standard deviation.

### Definition of Covariates

Covariates in the regression model contained the age, sex, insured amount, urbanization level of enrollee’s residential area, and former comorbidities. Regarding insured amount, it was calculated from the patients’ average monthly income and thus, also served as an economic index. Insured amount were transformed to ordinal variables according to the ranges of ≤ New Taiwan Dollar (NTD) 17,800, NTD 17,881–43,900, and ≥ NTD 43901. The urbanization level was classified as urban (levels 1–2), suburban (levels 3–4) and rural (levels 5–7) according to previous studies (Liu et al., 2006), with lower levels indicating greater urbanization. The effects of baseline comorbidities were evaluated on the basis of medical records during the year preceding entry into the cohort, using the Charlson-Deyo Comorbidity Index (CCI), which assessed the burden of comorbid conditions on a scale of 1–6 (Deyo et al., 1992). It is a commonly used method of categorizing comorbid conditions of patients based on the diagnostic codes found in the administrative dataset. Higher scores on the CCI were indicative of more severe impacts of concomitant comorbidities.

### Statistical Model

All statistical analyses were carried out using SAS Version 9.3 (SAS Institute Inc. Cary, NC, United States). Distributions of sociodemographic data and comorbidities between the CHM users and non-CHM users were compared using standardized differences. In contrast to the conventional method, this approach is often used for comparing baseline covariates in clinical trials as well as propensity-score matched studies because it appears to be not subject to the influence of sample size while assessing the balance in baseline variables between the two groups compared (Austin, 2008). A standardized difference of 0.1 or more was considered indicative of imbalance. Thereafter, to assess the independent effect of CHM use on the risk of RA, we conducted a Cox proportional hazards regression to calculate the adjusted HR with 95% confidence interval (CI), after adjusting for age, sex, income, urbanization level, and CCI simultaneously in the model. To further test the robustness of the relation of CHM use with RA risk, CHM users were divided into three subgroups according to the length of CHM use: 31–365 days, 366–730 days, and more than 730 days. The Kaplan-Meier method and log-rank test were used to estimate the event-free survival rate and to examine differences in the risk of RA across the four groups. Log (−log [survival]) versus log of survival time plot was inspected to verify the proportional hazards assumption. A *p* < 0.05 was considered statistically significant.

## Results

Of the whole study cohort, the CHM user and non-CHM user groups contributed data on 13,440 patients each. The mean age of patients was 52.10 ± 16.31 years, with female predominance (57.7%). Most of the enrollees had monthly incomes of NTD 17,881–43,900 (49.4%) and lived in urbanized areas (56.2%) (Table 1). The mean of CCI among them was 6.32 (±9.09). Collectively, there were no differences between the CHM users and non-CHM users in the baseline demographic data and comorbidities after the matching procedure (Table 1).

**FIGURE 2:**
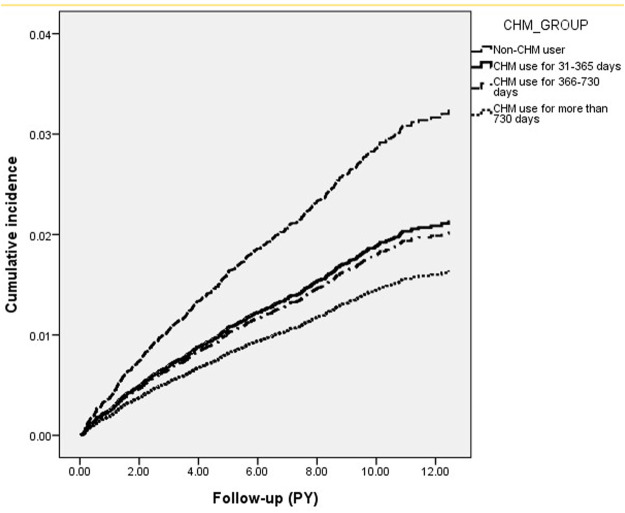
Cumulative incidence of RA across four groups (Log-rank test, *p* < 0.001).

Review of the whole study cohort identified 573 cases of RA, 359 in CHM non-users and 214 in CHM users, during follow-up periods of 123,089 and 111,661 PY, respectively. The incidence of RA was lower in CHM users than in non-CHM users (1.92 vs. 2.92 per 1000 PY) (adjusted HR = 0.63; 95% CI: 0.54–0.75) (Table 2). Notably, subgroups with longer duration of CHM use had lower risks of RA, with a dose-dependent relationship identified between days of CHM usage and RA risk. Specifically, the adjusted HR were 0.65 (95% CI = 0.54–0.77), 0.61 (95% CI: 0.41–0.91), and 0.51 (95% CI: 0.29–0.88) for patients with CHM use of 31–365 days, 366–730 days and >730 days, respectively (*p* for trend< 0.001) (Table 2). Results of Kaplan-Meier survival analysis and the log-rank test also showed differences in the RA-free survival rate across the four groups during the follow-up period (*p* < 0.001) (Figure 2).

**TABLE 2:** RA incidence (per 1000 PY) and RA risk in asthma patients with and without CHM use.

Patient group	Case	PY	Incidence	Crude HR (95% CI)	Adjusted HR^a^ (95% CI)
Non-CHM users	359	123,089	2.92	1	1
CHM users	214	111,661	1.92	0.65 (0.54–0.76)	0.63 (0.54–0.75)
CHM use for 31–365 days	175	88,763	1.97	0.66 (0.55–0.79)	0.65 (0.54–0.77)
CHM use for 366–730 days	26	14,292	1.82	0.62 (0.42–0.91)	0.61 (0.41–0.91)
CHM use for >730 days	13	8,606	1.51	0.52 (0.30–0.90)	0.51 (0.29–0.88)

a Model adjusted for age, sex, urbanization level, monthly income, and CCI, score.

RA: rheumatoid arthritis; CHM: Chinese herbal medicine; PY: person-year; HR: hazard ratio; CI: confidence interval.

Furthermore, as systemic and inhaled steroid are often prescribed to patients with asthma, we conducted a sensitivity analysis taking into account the co-medication to determine if this relation is robust, which included budesonide, fluticasone, beclometasone, ciclesonide, formoterol, and budesonide, betamethasone, dexamethasone, paramethasone, methylprednisolone, prednisolone, triamcinolone, hydrocortisone, and cortisone. All enrollees were divided into two groups based on if they received any one of these medications for more than 28 days after asthma onset (Lee et al., 2020). The proportion of the co-medication was similar between CHM users and non-CHM users (81.9% vs. 82.7%), and so the co-medication was unlikely to introduce remarkable confounding to our study. Consequently, we found the HR (0.69; 95% CI = 0.57–0.82) associated with CHM after adjusting for the co-medication remained statistically significant in the multivariate sensitivity analysis.

**FIGURE 3:**
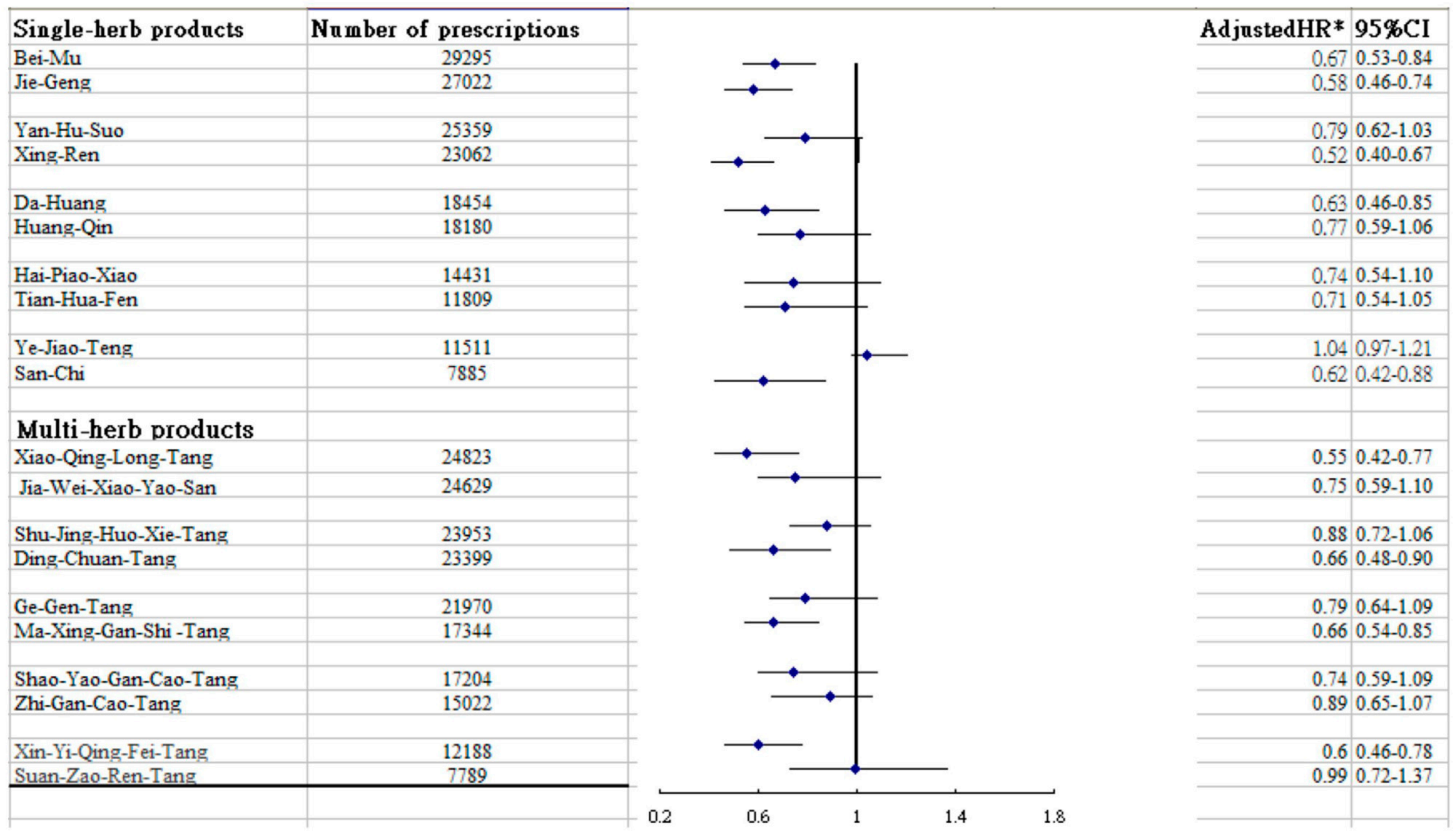
Risk of RA in relation to the 10 most-used single-herb and multi-herb CHM products for asthma patients.

Regarding the analysis stratified by age and sex, the benefit of CHM therapy in reducing the incidence of RA was more predominant among females regardless of age, with an adjusted HR of 0.58 (95% CI: 0.47–0.69) (Table 3). The most commonly prescribed CHM formulae are summarized in Table 4. Among them, nine CHM products were associated with a lower risk of RA, namely Xiao-Qing-Long-Tang, Ma-Xing-Gan-Shi-Tang, Ding-Chuan-Tang, Xin-Yi-Qing-Fei-Tang, Bei Mu, Jie Geng, Xing Ren, Da Huang and San Chi (Figure 3).

**TABLE 3:** RA incidence (per 1000 PY) and RA risk in asthma patients with and without CHM use, stratified by sex and age.

Age (years)	Non-CHM users			CHM users			Crude HR (95%CI)	Adjusted HR (95%CI)
	Case	PY	Incidence	Case	PY	Incidence		
Male								
≤50	19	25,540	0.74	14	19,919	0.70	0.94 (0.47–1.84)	0.92^a^ (0.41–1.64)
>50	55	30,558	1.80	43	27,386	1.57	0.87 (0.57–1.26)	0.84^a^ (0.56–1.26)
All	74	56,098	1.32	57	47,305	1.20	0.90 (0.63–1.24)	0.88^b^ (0.61–1.21)
Female								
≤50	102	27,895	3.66	63	28,973	2.17	0.59 (0.43–0.80)	0.58^a^ (0.43–0.79)
>50	183	39,096	4.68	94	35,384	2.66	0.57 (0.43–0.71)	0.56^a^ (0.43–0.71)
All	285	66,991	4.25	157	64,357	2.44	0.57 (0.46–0.69)	0.58^b^ (0.47–0.69)

a Model adjusted for urbanization level, monthly income, and CCI, score.

b Model adjusted for age, urbanization level, monthly income, and CCI, score.

RA: rheumatoid arthritis; CHM: Chinese herbal medicine; PY: person-years; HR: hazard ratio; CI: confidence interval.

**TABLE 4:** Risk of RA in relation to the 10 most-used single-herb and multi-herb CHM products for asthma patients.

Chinese herbal product	Ingredients or generic name	Functional classification
Single-herb products		
Bei Mu	Fritillariae Thunbergii Bulbus	Eliminate phlegm by cooling, moisten lung to arrest cough, and remove stasis to reduce swelling
Jie Geng	Radix Platycodi	Support respiratory health and benefit the throat; contain anti-inflammatory, antibacterial, expectorant and immune boosting properties
Yan Hu Suo	Corydalis yanhusuo	Used to treat Qi stagnation, blood stasis, chest pain, abdominal pain, amenorrhea, dysmenorrhea, and postpartum stasis
Xing Ren	Semen Armeniacae	Used to relieve cough, expel phlegm and ease breathing
Da Huang	Rheum officinale	Address constipation, and other inflammatory issues in the colon, liver, gallbladder, stomach, and reproductive organs
Huang Qin	Scutellaria baicalensis	As an adjuvant therapy of inflammation, diabetes, hypertension, different kinds of cancer and virus related diseases
Hai Piao Xiao	Endoconcha Sepiae Os Sepiae seu Sepiellae	Control acidity, harmonize the stomach, and alleviate pain
Tian Hua Fen	Radix Trichosanthis	Clear lung heat, dissolve phlegm, relieve toxicity, and expel pus
Ye Jiao Teng	Caulis Polygoni Multiflori	Nourish the Heart and Liver Blood and expel Wind in the collaterals to stop itch and treat skin disorders
San Chi	Panax	Promote blood circulation, stop bleeding, and replenish blood
Multi-herb products		
Xiao-Qing-Long-Tang	Herba Ephedrae, Rhizoma Zingiberis, Ramulus Cinnamomi, Radix et Rhizoma Asari, Rhizoma Pinellia, Fructus Schisandrae Chinensis, Radix et Rhizoma Glycyrrhizae, and Radix paeoniae alba	Used to treat bronchial asthma and allergic rhinitis
Jia-Wei-Xiao-Yao-San	Bupleurum Root, Chinese Angelica Root, White Peony Root, White Atractylodes Rhizome, Poria, Licorice Root, Moutan Bark, Gardenia Fruit, Mint Herb, Ginger	Used to treat functional dyspepsia
Shu-Jing-Huo-Xie-Tang	Tang-kuei root, White peony root, Corydalis root, Chin-chiu, Cnidium root, Raw rehmannia root, Peach kernel, Hoelen fungus, Atractylodes root, Citrus peel, Notopterygium root, Fragrant angelica, Scabrous gentiana root, Fang feng root, *Achyranthes* root, Ginger root, Chinese licorice root	Clear Heat, cool the Blood, nourishes Yin and generates fluids; breaks up Blood Stasis and invigorate Blood circulation; Strongly dry Dampness, tonify the Spleen, induce sweating and expels Wind-Dampness; promote urination and leach out Dampness
Ding-Chuan-Tang	Herba Ephedrae, Semen Ginkgo, Flos Farfarae, Rhizoma Pinellia, Semen armeniacae amarum, Fructus Perilla, Cortex Mori, Radix Scutellariae, and Radix et Rhizoma Glycyrrhizae	Applied mainly for patients with coughing, wheezing, chest tightness, and asthma
Ge-Gen-Tang	Puerariae radix (Pueraria lobata Ohwi), Ephedrae Herba (Ephedra sinica Stapf), Cinnamomi Ramulus (Cinnamomum cassia Blume), Paeoniae Radix (Paeonia lactiflora Pallas), Glycyrrhizae Radix preparata (Glycyrrhiza uralensis Fischer), Zingiberis Rhizoma (Zingiber officinale Roscoe), and Zizyphi Fructus (Ziziphus jujuba Mill. var. inermis Rehder)	Induce sweating to release the exterior symptoms and dispel Wind-Cold; is indicated for stiff neck, headache, muscle aches, alternating chills and fever, sneezing, cough, nasal congestion, and runny nose
Ma-Xing-Gan-Shi -Tang	Forsythia, Honeysuckle, Ephedra (Branch), Bitter Almond (Fried), Gypsum, Banlangen	Present a prominent antivirus effect and is often used to treat pulmonary diseases
Shao-Yao-Gan-Cao-Tang	Rx. Paeoniae Alba, Rx. Glycyrrhizae Preparata	Antioxidative and antiaggregation effect
Zhi-Gan-Cao-Tang	Licorice Root, Ginger, Rehmannia, Cinnamon Twig	Benefit the Spleen, nourish the Heart, nourish Blood, calm the Shen, and moderate and harmonize the harsh properties of other herbs
	Ophiopogon Root, Cannabis Seed, Jujube Fruit, Ginseng Root, Ass Hide Gelat
Xin-Yi-Qing-Fei-Tang	Gypsum, Ophiopogon Tuber, Scutellaria Root (SR, root of Scutellaria baicalensis), Gardenia Fruit, Anemarrhena Rhizome, Lilium Bulb, Magnolia Flower, Loquat Leaf, and Cimicifuga Rhizome	Used to treat sinusitis associated with purulent nasal discharge and reddish nasal mucosa
Suan-Zao-Ren-Tang	Ziziphi Spinosae Semen, Poria, Chuanxiong Rhizoma, Anemarrhenae Rhizoma, and Glycyrrhizae Radix Et Rhizoma	Used to treat insomnia and anxiety

## Discussion

Reducing the risk of RA following asthma onset is an important issue. The present study is probably the first to clarify the association between CHM use and risk of RA amongst asthmatic patients. The results showed that, compared with those without CHM therapy, asthmatic patients treated with CHM had a 37% lower risk of developing RA. Furthermore, those receiving CHM treatments for more than 2 years had a nearly 50% lower risk of RA compared to non-users. The dose-dependent response implies a causal relation. No data available from previous research have shown this relationship, rendering a direct comparison of results impossible. Yet, the findings obtained herein are consistent with earlier research findings on other outcomes and add to the growing body of knowledge on the beneficial effects of CHM for patients with chronic diseases (Chan and Ng, 2020; Li H.-H. et al., 2021).

Our study found that female patients benefited more than male patients from receiving CHM treatment, with a 42% lower risk of RA, which is compatible with an earlier report (Li KH. et al., 2021). We infer that women possess better health consciousness and thus are more likely to seek medical care in the early stage and comply with the prescribed medical regimen (Shih et al., 2012). Hormones, especially estrogen, may also account for the difference. Estrogen has been found to affect the expression of inflammatory mediators, such as interleukin (IL)-1, IL-6 and tumor necrosis factor-α (TNF-α) (Shivers et al., 2015), all of which were reported to play decisive roles in the pathogenesis of RA (Malemud, 2013).

Of particular note, this work is to specify the herb products that may serve as potential therapeutic agents to prevent RA. Among the multi-herb products commonly used to treat asthma, we identified four herb formulae that might reduce the risk of RA. Among them, Xiao-Qing-Long-Tang and Xin-Yi-Qing-Fei-Tang were found to lower the risk of developing RA by over 40%. In traditional Chinese medicine, both formulae are designed to lower the adverse flow of qi by warming the lungs (Lu et al., 2020). Studies using an animal model found that the administration of either Xiao-Qing-Long-Tang or Xin-Yi-Qing-Fei-Tang could similarly modulate the expression of IL-6 and TNF-α by suppressing the pivotal activities of nuclear factor kappa-light-chain-enhancer of activated B cells (NF-κB) (Kim et al., 2009; Luo et al., 2019). At present, converging scientific consensus agrees that, upon activation, NF-κB promotes Th cell differentiation by regulating T cell receptor signaling, as well as functioning, in innate immune cells to mediate the induction of inflammatory mediators, thus driving the risk of autoimmune disease (Liu et al., 2017). Echoing the results reported in a previous investigation, our study revealed that Ma-Xing-Gan-Shi-Tang was one of the frequently used Chinese herbal products for treating asthma (Song et al., 2018). This compound has been found to improve lung function by reducing the levels of cyto-inflammatory factors such as TNF-α and other inflammatory genes *via* regulation of the PI3K-Akt signaling pathway (Song et al., 2018). This pathway has been found to be able to promote the aggressiveness of immune cell and synoviocyte proliferation, leading to susceptibility to RA (Malemud, 2013). These findings may explain why the use of Ma-Xing-Gan-Shi-Tang may decrease the risk of RA. Another herbal product shown to be effective in lowering RA risk is Ding-Chuan-Tang. Using a rodent model, a study showed that this formula can prominently suppress eosinophil infiltration in the lung, airway hyperresponsiveness, and Th2 cell-associated cytokine expression in bronchoalveolar lavage fluid by reversing the inflammation-immune system imbalance (Ma J. et al., 2021), thereby reducing, to some extent, the risk of RA.

Regarding the single-herb products used to treat people with asthma, we noted that Xing Ren was associated with a reduced risk of RA. In one study, rats treated with Xing Ren had markedly reduced secretion of inflammatory cytokines, through inhibition of the NF-κB and mitogen-associated kinase (MAPK) signaling pathways (Wang YT. et al., 2020). The latter pathway is involved in a diverse array of cellular processes that include inflammation, angiogenesis, and proliferation (Thalhamer et al., 2008). Our study also delineated the effect of Bei Mu together with Da Huang on the incidence of RA. In studies of both humans and animals, these prescriptions exerted a potent anti-arthritic effect by inhibiting the production of inflammatory mediators such as TNF-α, IL-6, and IL-8 (Liao et al., 2021; Li et al., 2019; Shrimali et al., 2013). Systemic chronic inflammation has been hypothesized as the most important mechanism triggering RA onset (Malemud, 2013). Jie Geng was also found to be associated with a lower risk of developing RA in patients with asthma. In traditional Chinese medicine, Jie Geng is frequently used to reduce cough and expectorate. Modern pharmacological research has shown that Jie Geng may dose-dependently reduce nitric oxide production in lipopolysaccharide-stimulated macrophage RAW264.7 cells and reduce the levels of IL-6 and TNF-α inflammatory factors (Ma X. et al., 2021). As to San Chi, it was proposed to have anti-metastatic and anti-inflammatory activities through the regulation of matrix metalloproteinase (MMP) *via* modulation of various cellular MAPKs and/or Akt signaling pathways (Lee, 2021). The activation of these pathways may be related to synovial hyperplasia and MMP gene expression, which may further degrade the collagen matrix components of joints to increase the susceptibility to RA (Itoh et al., 2002; Malemud, 2013).

The database used in this study has several strengths, including its large sample size, the use of electronic records from a national health insurance registry, and a uniform approach to outcome assessment, all of which strengthen the validity of the study findings. In addition, selection bias is minimal, because the NHI database includes over 99.9% of residents in Taiwan. Furthermore, to minimize confounding, we conducted 1:1 propensity score matching for age, sex, monthly income, residential area, comorbidities, and follow-up time. Nonetheless, several limitations of this study merit attention. First, our findings were derived from analysis of retrospective cohort data based on ICD-9-CM diagnostic codes. Thus, some relevant cases may have been misclassified. To improve the accuracy of case identification, we enrolled only those patients with new-onset asthma or RA, requiring either: 1) at least three outpatient visits with consistent diagnoses or 2) at least one inpatient admission. In addition, the diagnosis of RA was further confirmed by catastrophic illness certification by the NHI. The NHI of Taiwan also randomly reviews the charts and audits medical charges to verify the accuracy of claims and imposes heavy penalties for malpractice. Furthermore, the misclassification was expected to occur at random, which would shift the HR towards the null value and thus provide a more conservative estimate of effect. Second, the LHID did not contain information on genetic and environmental risk factors of RA such as family history. So residual confounding might occur in the observed association because participants were not initially randomly assigned into users and non-users. A randomized controlled trial to validate the findings is warranted.

## Conclusion

Individuals with asthma suffer from autoimmune rheumatic diseases more frequently than the general population, including RA. This population-based study found that the integration of CHM into asthma treatment regimen was associated with a lower risk for developing RA with a dose-dependent relationship in terms of the duration of prescription, which suggests a causal relationship. This novel finding can provide an impetus for further clinical and mechanistic studies and paves the way for further personalized therapies with the use of adjunctive CHM for the management of other pulmonary disorders.

## Data Availability

The datasets analyzed in this article are not publicly available. Data are available from the National Health Insurance Research Database (NHIRD) published by Taiwan National Health Insurance (NHI) Bureau. Due to legal restrictions imposed by the government of Taiwan in relation to the “Personal Information Protection Act,” data cannot be made publicly available. Requests to access the datasets should be directed to the NHIRD and the corresponding authors.

## Abbreviations

CHM, Chinese herbal medicine; CI, confidence interval; HR, hazard ratio; ICD-9-CM, International Classification of Diseases, Ninth Revision, Clinical Modification; IL, interleukin; LHID, Longitudinal Health Insurance Database; MAPK, mitogen-associated kinase; MMP, matrix metalloproteinase; NF-κB, nuclear factor kappa-light-chain-enhancer of activated B cells; NHI, National Health Insurance; NTD, New Taiwan Dollars; PY, person-year; RA, rheumatoid arthritis; TNF-α, tumor necrosis factor-α.
